# The CRISPR/Cas9 System and the Possibility of Genomic Edition for Cardiology

**DOI:** 10.5935/abc.20160200

**Published:** 2017-01

**Authors:** Marcela Corso Arend, Jessica Olivaes Pereira, Melissa Medeiros Markoski

**Affiliations:** 1 Laboratório de Cardiologia Molecular e Celular - Programa de Pós-Graduação em Ciências da Saúde (Cardiologia) - Instituto de Cardiologia/Fundação Universitária de Cardiologia - IC/FUC, Porto Alegre, RS - Brazil; 2 Universidade do Vale do Rio dos Sinos - UNISINOS, São Leopoldo, RS - Brazil; 3 Universidade Federal de Ciências da Saúde de Porto Alegre - UFCSPA, Porto Alegre, RS - Brazil

**Keywords:** Cardiovascular Diseases / mortality, Morbidity, Risk Factors, Prevention & Control, Molecular Biology, Genomics

## Introduction

Cardiovascular diseases (CVD) and their associated pathologies are among the greatest causes of morbidity and mortality, entailing approximately 17.3 deaths a year.^1^ This class of pathology as a whole has a multi-factor etiology. Its possible prognoses lead to public health issues, with its incidence being related to behavioral, metabolic and genetic risk factors.^2^ In spite of the fact that the treatments established for the CVDs and their possible prognoses decrease the rhythm of progression of the illness, the need to develop therapeutic approaches able to reverse the pathology and its complications is growing.

The progresses in the fields of molecular and cellular biology have allowed the elucidation of molecular pathways and genetic causes involved in the establishment and progression of the CVDs, outlining a new viewpoint with regard to the prevention, treatment and possible outcomes of this pathological class. Recent discoveries, both experimental and those obtained by means of bioinformatics tools, regarding the molecular bases of cardiovascular dysfunctions, have been pointing to considerable therapeutic targets.^3^ However, most of these targets cannot be pharmacologically manipulated, which makes them potential candidates for genic therapy, such as the factors involved, for example, in angiogenesis, apoptosis and endothelial dysfunction.^4^ Within such context, gene manipulation may help suppress genetic factors connected to the incidence of the CVDs, as well as to mitigate the clinical complications caused by ischemic and occlusive events. Thus, the development and improvement of genome edition tools allow the creation of therapies focused in the genetic risk factors to cardiovascular damage and fundamental morphophysiological issues caused by the CVDs. In such context, the system formed by clustered regularly interspaced short palindromic repeats (CRISPR), and its CRISPR associated protein-9 (Cas9), stands out due to how easy it is to use it, its high specificity, easy in vitro and in vivo manipulation, in addition to the possibility of simultaneously editing multiple targets. Given the genomic complexity that intervenes in the CVDs, we shall indicate herein certain possibilities of applying the CRISPR/Cas9 tool in Cardiology.

### Unraveling the CRISPR/Cas9 system

**Figure 1:**
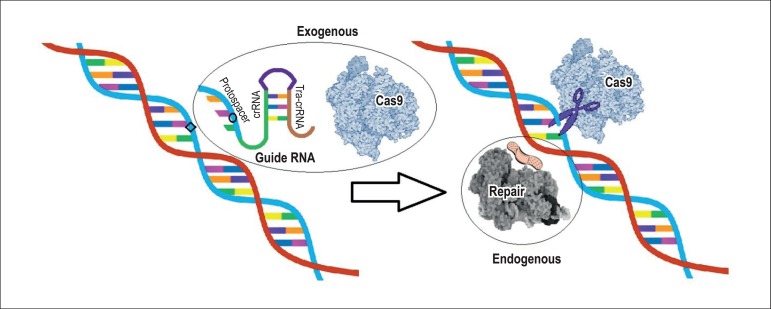
CRISPR/Cas9 system - target recognition mechanism. The guide RNA is designed to recognize the target sequence to be modified in the RNA and introduce modifications. When the pairing of nitrogenized bases occurs (due to the annealing of the target sequence with the region of the guide RNA protospacer), some modifications are added (represented hereby the circle) and the Cas9 enzyme is activated, causing breaks to the DNA double strand (where there are pairing flaws due to the mutations introduced). The breaks activate the intracellular repair systems that remake the double strand, accepting the modifications from the guide RNA. The new mutations generally cause flaws in the sequence and generate non-functional proteins. But the mechanism can also be used to correct mutations originally present in the DNA and generate functional proteins.

Developed from molecular organisms of the bacterial immune system, the CRISPR system allows the edition of the genome by means of splicing of the DNA by an endonuclease (Cas9), guided based on an RNA sequence, which is able to pair up with the bases of a target sequence (Figure 1).^5^ The CRISPR genetic structure, in the bacterial system, is made up of clustered regularly interspaced short palindromic repeats. The repeats and the spacers (which may contain interspacing viral sequences), when transcribed, form the transactivator RNA (or guide RNA), which serves to direct the Cas9 enzyme, a nuclease, to the target (in this case, the parasite virus sequence). Taking advantage of this strategy, both the Cas9 protein and the guide RNA can be introduced in vitro into other cells and directed to specific places in the genome, for them to cause breaks to the double strand. After this splicing, the intrinsic molecular machinery of the organism, responsible for the correction of errors in the genome, is used to alter the DNA sequence, adopting the modification. The system can thus be used both to repair mutations (restoring genic function) and to introduce new mutations (causing the genic "knockout"). Therefore, by conciliating sophisticated molecular and biotechnological techniques, the CRISPR/Cas9 system was proposed for application on genomic editing and is currently commercially available for thousands of targets.^6^ Both the RNA and the Cas9 protein, produced in vitro, can be delivered to the cells using different mechanisms, such as the use of vectors or chemical agents.

The most simple applicability of the CRISPR system is connected to changing single or certain bases in genes with a well-defined allelic relationship. It is important to stress that this relationship of Mendelian dominance must be taken into account for the genic function to be achieved, both to activate it and to inhibit it. However, bi-allelic modifications have also been successfully obtained^7^. Moreover, the use of the CRISPR/Cas9 has also been proposed for embryonic stage in animal models, where the progeny can generate "founding" organisms (by recombination) containing allelic mutations that lead to the "knockout" effect or with diminished expression.^8^ In such context, the CRISPR/Cas9 system is being quickly adopted to edit and modify genomes in several cellular types, including stem cells,^9^ and has been giving good results in the edition of human genes.^10^ The press recently reported that researchers from the University of Pennsylvania have been given approval from the Food and Drug Administration (FDA) to conduct a clinical study to begin in 2017, the targets of which are 3 genes involved in cancer. Thus, the question arises regarding the possibility of applying the CRISPR/Cas9 system to such a biologically complex situation as cardiovascular diseases.

### How to use the CRISPR/Cas9 system in Cardiology

The first step to suggest the use of the CRISPR/Cas9 system for a certain CVD must be based on an in-depth study of the potential molecular targets involved in the disease. In such scenario, the use of bioinformatics tools and genic sequence banks available online (such as the National Center for Biotechnology Information, NCBI; and the DNA Data Bank of Japan, DBDJ) and of predicted proteins (such as the Universal Protein Resource, UniProt, allocated to the European Molecular Biology Laboratory, EMBL), in addition to single polymorphism banks, SNP (http://www.ncbi.nlm.nih.gov/snp), may assist with the process. Once the targets have been chosen, a detailed analysis of the function of the exons (codification sequences of the genes) must also be carried out. In possession of every information necessary, the guide RNA may be designed and commercially acquired. There are currently several research laboratories that are using the CRISPR/Cas9 tool to edit genes involved in CVD and testing them on cellular systems, conducting pre-clinical trials and scheduling clinical studies. Even though the cardiovascular context is complex, some pathologies are more or less connected to certain genic products, the interaction of which with other molecules is known, as described below, facilitating the feasibility of using the CRISPR/Cas9 system.

One of the great issues with the maintenance of the coronary artery disease (DAC) is the elevation of the LDL, where pharmacological intervention seeks to decrease it by using statins. Given that some patients are intolerant to such substance or do not respond well to it, several researches are being carried out, focusing on inhibiting the Proprotein convertase subtilisin/kexin type 9 (PCSK9), which helps degrade the LDL receptors, which causes an increase to the level of lipoprotein in the blood stream. Through the CRISPR/Cas9 system, Ding et al (2014) introduced a *loss of function* for the PCSK9 gene in the livers of mice, using adenoviruses as "vehicles", and showed a decrease of the cholesterol levels by over 40%.^11^ In a study with rabbits, also focused on decreasing the progression of the atherosclerotic plaque, "knockout" animals were developed by genomic edition, by inhibiting several genes, such as Apolipoprotein E (ApoE), CD36, the LDL receptor, leptin, ryanodine receptor type 2 (RyR2), among others.^12^ These studies show that the CRISPR/Cas9 system is viable to alter the function of genes connected to CVDs. This favors the exploration of the use of the molecular tool for other mechanisms concerning CVDs.

A good study target for a possible use of the CRISPR/Cas9 is the β-adrenergic system, one of the systems responsible for vasoconstriction/vasodilation and maintenance of the blood pressure and heart rate. Add to that the fact that the renin-angiotensin-aldosterone system also has a crucial role in maintaining the hemodynamic stability. Both systems are regulated by an extensive effector network, such as hormones and peptides, receptors, kinase proteins and other enzymes, working both in outside and inside the cells. In this regard, it would be very interesting to test and appraise the genomic edition tool to assist with the systemic arterial hypertension treatment.

Our group, concurrently with the application of alternative therapies, such as cellular and genic therapy, to treat CVDs, developed the first clinical study in the country to promote angiogenesis, by exogenous expression, by administering a plasmid containing the cDNA related to the vascular endothelial growth factor (VEGF) in patients with refractory angina, showing that the technique is safe and improves the ventricular ejection fraction.^13^ We are currently focusing our efforts on understanding mechanisms that may help with the interventions (surgical, pharmacological, dietetic, etc.) for CVDs, especially for dilated cardiomyopathy (DCM) and ischemic caridiopathies.^14^ In collaboration with researchers from the Cancer Institute, we are using the CRISPR/Cas9 system to achieve the inactivation of the function of a tissue-specific kinase MAP, coded by gene TNNI3K, which interact with cardiac troponin I and, when exacerbated, causes a progression of the DCM, leading to heart failure and increasing the risk of death.^15^

Not just the inhibition context, but also the possibility of edition to activate genes in order to stimulate functions connected to, for example, the survival of cardiomyocytes in the after-infarction period, inducement of homing (migration, proliferation and differentiation of stem cells), increase of the level of anti-inflammatory cytokines and metalloproteinases inhibitor proteins (which lead to the pathological ventricular remodeling), in addition to other mechanisms, may be explored with regard to the CVDs. However, due to the multi-factor conditions attributed to the etiology and prognosis of this class of pathologies, the clinical transposition of results obtained through molecular analyses in in vitro cellular systems or animal models, just as with other more innovative approaches, is still a challenge.

In most cases, genetic, environmental and behavioral factors work together to entail a CVD. Even though, in certain cases, likely factors of prediction of the outcomes are observed, it is not yet possible to accurately forecast the influence of the activation/inactivation of genes in relation to the clinical statuses. Lastly, just as with any new technology, the risks, physiological adaptations, implications of the immune response and maintenance of homeostasis, which may be modulated by the CRISPR/Cas9 system, need to be very well assessed. But the possibility of using the new molecular tool in Cardiology can be glimpsed and perhaps, in the near future, come to benefit the population's health.
